# ASM in Alzheimer's disease

**DOI:** 10.18632/oncotarget.6333

**Published:** 2015-11-14

**Authors:** Jong Kil Lee, Hee Kyung Jin, Jae-sung Bae

**Affiliations:** Stem Cell Neuroplasticity Research Group, Cell and Matrix Research Institute, Kyungpook National University, Daegu, Korea

**Keywords:** acid sphingomyelinase, alzheimer

A remarkable rise in life expectancy and the declining prevalence of certain causes of late-life mortality allow dementia to become a common occurrence with age. Alzheimer's disease (AD) is the most prevalent form of dementia characterized clinically by progressive loss of memory, and pathologically by the presence of neuritic plaques and neurofibrillary tangles [1]. Many scientists effort to understand the AD pathogenesis by investigating a complex interaction between genes and the environment. There are profound biochemical alterations in multiple pathways in the AD brain, including changes in amyloid-β (Aβ) metabolism, tau phosphorylation, lipid regulation, and cellular disposal system, although to date the underlying mechanism(s) leading to these complex abnormalities, remain largely unknown [1-3]. Acid sphingomyelinase (ASM, EC 3.1.4.12) is a key enzyme that regulates tissue homeostasis by affecting diverse signaling process, including cell survival, proliferation, and differentiation, but also in senescence and apoptosis [2, 4]. In the brain, this enzyme normally works in harmony with other sphingolipid-metabolizing enzymes and sphingolipid metabolites for proper neuronal function. Therefore, the subtle changes in sphingolipid homeostasis by ASM are intimately involved in neurodegenerative diseases including AD [4]. However, the role of ASM in AD and the cellular mechanisms that link ASM and AD have not been fully characterized.

In a recent study published in Journal of Experimental Medicine, we reported that increased ASM activity in AD results in defective autophagy due to disruption of lysosomal biogenesis [5]. ASM activity was significantly increased in plasma and fibroblast samples derived from AD patients compared with that of normal aged individuals [5]. ASM was not elevated in samples from Parkinson's disease patients. These results confirmed ASM activity is a specific signature of AD and influence disease progression and/or pathogenesis. To address the influence of ASM on AD pathology, amyloid precursor protein/presenilin 1 (APP/PS1) double mutant and APP/ PS1/ASM^+/−^ triple mutant mice were used. Similar to AD patients, ASM activity was elevated in plasma, fibroblast, and brain (especially neurons) of APP/PS1 mice. This activity was decreased in APP/PS1/*ASM^+/−^* mice to levels within the normal range or lower. Strikingly, AD pathology including Aβ deposition and memory dysfunction was significantly reduced in APP/PS1/ASM^+/−^ mice [5].

There is growing evidence to suggest that dysfunction of the proteolytic degradation system could affect AD pathogenesis and lead to enhanced Aβ deposition. Autophagy, a process to destroy misfolded proteins or breakdown faulty components and then recycle, is known to be markedly impaired in AD [3]. We found that APP/PS1 mice showed an increased autophagosome, but APP/PS1/*ASM^+/−^* mice revealed a reduced these vesicles, indicating that elevated ASM activity in AD causes autophagy dysfunction and that partial genetic inhibition of ASM leads to restoration of autophagy process. Similarly, the inhibitory effect of ASM in pathogenesis of AD also has been confirmed in amitriptyline-hydrochloride (a known inhibitor of ASM, AMI) treated APP/PS1 mice [5].

To examine the direct relationship of ASM and autophagic dysfunction, we analyzed recombinant ASM treated human neuron and fibroblast. We found that increased lysosomal ASM induces autophagosome accumulation in a concentration dependent manner. We also identified that ASM causes a defect of autophagic degradation, but not induction [5]. Recently, the transcription factor EB (TFEB) was identified as a master regulator of the autophagy-lysosome pathway (ALP) and lysosome biogenesis. Enhancement of TFEB function is able to stimulate ALP function and promote misfolded protein clearance [6]. We identified that lysosomal ASM acts as an inhibitor of autophagic protein degradation by reducing ALP function and lysosome biogenesis [5].

To explore whether the observed effects of ASM *in vitro* and *in vivo* results are paralleled by similar alterations in patients, human AD fibroblasts (PS1-FAD and apoE4) and induced pluripotent stem cells-derived neurons with PS1 mutation were analyzed. We found that abnormal autophagy observed in AD mice by ASM also occurs in AD patient-derived fibroblast and neuron, and restoration of ASM back to normal levels is able to ameliorate autophagic dysfunction by restoring lysosomal biogenesis in AD patient cells.

The combination of the vast knowledge of lipids and their biophysical and biochemical properties is translated into the development of new therapeutic and diagnostic modalities for diverse diseases. Similarly, the field of sphingolipids becomes very active and popular as a result of the understanding of sphingolipids' role in disease environment. This dramatic increase in the understanding of the various functions of sphingolipids leads to the development of several novel drugs, the first of which, a sphingosine-1-phosphate agonist for multiple sclerosis, was recently approved by the Food and Drug Administration [7]. The results of our study show that increased ASM activity in AD contributes to the pathology through abnormal lysosomal/autophagic process and restoration of ASM effectively blocks AD progression by recovering autophagic process. Collectively, our findings may open up new exciting possibilities for the drug development of AD and other neurodegenerative diseases caused by lysosomal dysfunction.
